# Quantifying Tip60 (Kat5) stratifies breast cancer

**DOI:** 10.1038/s41598-019-40221-5

**Published:** 2019-03-07

**Authors:** A. McGuire, M. C. Casey, A. Shalaby, O. Kalinina, C. Curran, M. Webber, G. Callagy, E. Holian, E. Bourke, M. J. Kerin, J. A. L. Brown

**Affiliations:** 1 Discipline of Surgery, School of Medicine, Lambe institute for Translational Research, National University of Ireland, Galway, Ireland; 2 School of Mathematics, Statistics and Applied Mathematics, National University of Ireland, Galway, Ireland; 3 Discipline of Pathology, School of Medicine, Lambe institute for Translational Research, National University of Ireland, Galway, Ireland

## Abstract

Breast cancer is stratified into four distinct clinical subtypes, using three key biomarkers (Her2/Neu gene status, Estrogen and Progesterone receptor status). However, each subtype is a heterogeneous group, displaying significant variation in survival rates and treatment response. New biomarkers are required to provide more precise stratification of breast cancer cohorts to inform personalised treatment options/predict outcomes. Tip60 is a member of the MYST sub-family of histone acetyltransferases (HATs), and is directly involved in genome maintenance, gene regulation and DNA damage response/repair pathways (key chemotherapeutic influencing mechanisms). We aimed to determine if quantifying Tip60 staining patterns improved breast cancer stratification. We defined Tip60 protein *in vivo*, quantifying location (cytoplasmic, nuclear), percent of cells and staining intensity in a breast cancer tissue microarray (n = 337). A significant association of specific Tip60 staining patterns with breast cancer subtype, ER or PR status and Tumour grade was found. Importantly, low Tip60 mRNA expression correlated with poor overall survival and relapse free survival. We found Tip60 is a biomarker able to stratify breast cancer patients, and low Tip60 expression is a significant risk factor indicating a higher chance of disease reoccurrence. This work highlights Tip60 regulation as a key factor influencing the development of breast cancer.

## Introduction

Modifications to histones (acetylation, methylation, phosphorylation) regulate chromatin structure by opening or closing chromatin. Histone acetylation is required for many aspects of gene regulation, metabolism, and genome organization/maintenance^1–6^. Significantly, dysfunctional acetylation has been implicated in numerous diseases, including cancer^1,7,8^. Two opposing classes of enzymes regulate histone acetylation, histone acetyltransferases [HATs; also known as lysine (K) acetyltransferases (KATs)] and histone deacetylases [HDACs; also known as lysine deacetylases (KDACs)]. While HDAC inhibitors are in clinical trials for cancer treatment the therapeutic potential of hindering the opposing machinery, KATs, for the treatment of cancer has only recently been recognised^3,7,9^.

The KAT family consists of 17 members with several distinct sub-families of KATs, the largest and most diverse being the MYST family (including MOZ, YBF2, MOF and Tip60)^3,10^. In addition to a well-known role in histone acetylation, the MYST family has an increasing substrate range^11^. Within the MYST family the importance of Tip60 is highlighted, as a Tip60 knockout is lethal^12,13^. This essential role for Tip60 is further demonstrated in cancer cells, where down-regulation results in cell death^7,13,14^. Tip60 is encoded by the Kat5 gene (with 4 isoforms), producing a ~60 KDa protein with a histone acetyltransferase domain and chromodomain. Tip60 has many diverse substrates, which is reflected in its diverse role in cellular processes. These include DNA damage response, the cell cycle, apoptosis, signalling and transcriptional regulation^6,9,15,16^. A key role of Tip60 is its regulation of the DNA double stand break (DSB) response through acetylation, leading to activation of the key protein kinase ATM (ataxia telangiectasia mutated)^13^. The importance of Tip60-dependent activation of ATM following DSB is demonstrated by Tip60 knockdown, which inhibits the DSB response and induces cell sensitivity to ionizing radiation^15^.

Tip60 haploinsufficiency has been reported in human tumours including breast, leading to the proposal that Tip60 is a tumour suppressor^12^. Supporting this, a significant reduction in overall Tip60 staining has been observed in small cohorts of breast and prostate cancer samples^12,17^. Furthermore a significant reduction in Tip60 protein and mRNA expression has been found in several other cancers^3,6,7,9,10,16,18–23^. Combined, these results indicate that cancer cells require a minimum low level of Tip60 for survival, and that reduced levels of Tip60 may correlate with a worse prognosis.

Here we have quantified Tip60 cellular localisation and levels (protein and mRNA) *in vitro* (cell lines) and *in vivo* (tissue microarray and expression databases) in breast cancer. The *in vivo* results were then correlated with key clinically relevant clinicopathological benchmarks (including overall survival, relapse free survival, breast cancer subtype, receptor status). Here we report the value of quantifying Tip60 levels for the stratification of breast cancer.

## Material and Methods

### Cell culture conditions

Human breast cancer cell lines (MDA-MB-231, MDA-MD-468, MCF7, T47D, SK-BR3) were cultured at 37 °C and 5% CO2 in the appropriate media as defined by the ATCC using standard techniques. All cell lines were purchased from ATCC.

### Reverse Transcription Polymerase Chain Reaction (RT-PCR)

Total mRNA was extracted with Tri Reagent (Sigma) as per manufacturer’s instructions. cDNA was synthesized from the extracted mRNA using SuperScript (Life Technologies) as per manufacturer’s instructions. Equal amounts of cDNA were then used in a PCR reaction to amplify the n terminal regions of Kat5 and endogenous control gene GAPDH. cDNA synthesis and RT-PCR were performed using standard conditions, as per the manufactures instructions (Invitrogen).

### Western Blotting

Whole cell extracts were prepared from indicated cells lines by re-suspending cell pellets and incubating in Lysis Buffer (50 mM HEPES pH 7.5, 150 mM NaCl, 0.1% Tween-20, 1 mM EDTA, 10% glycerol, 1 mM DTT, 1 mM PMSF, 1 mM NaVO4, 1 mM aprotinin and 1 mM pepstatin) at 4 °C for 1 h. 30 μg of total protein was separated using SDS-PAGE gels and transferred to nitrocellulose membranes. Chemiluminescence was detected using SuperSignal West Pico Chemiluminescent Substrate® (Thermo Scientific) and medical X-ray film (Konica Minolta). Antibodies: Tip60 (K-17: sc-5727) Santa Cruz and beta-Tubulin (ab6046) Abcam.

### Subtypes definitions

Breast cancer molecular subtypes were defined based using standard accepted markers: Luminal A (ER and/or PR positive, HER2 negative); Luminal B (ER and/or PR positive, HER2 positive); HER2-overexpressing (ER and PR negative, HER2 positive); Triple negative (ER, PR and HER2 negative). The HER2 receptor status was identified by immunohistochemistry with any inconclusive results confirmed using FISH analysis.

### Tissue microarray (TMA)

Clinical breast tissue samples comprised core biopsies, wide local excisions and mastectomy specimens received by the Galway University Hospital pathology department (1999–2005) which were used to construct a consecutive tissue microarray, based on breast cancer diagnosis and availably of biopsy tissue in the paraffin block. Cores (0.6 mm diameter) of formalin-fixed paraffin-embedded (FFPE) tissue were used to construct the TMA, as previously described^24–27^. Tumour areas in each tumour block were identified by a clinical pathologist using haematoxylin and eosin (H&E) stain prior to core punching. Pathological data was collected from the clinical pathology reports for each patient. Images of Tip60 stained sections were captured using an Olympus VS120 Digital Scanner with a 40× objective and images processed using OlyVIA software (v2.8).

### TMA Patient cohort

This study group consists of consecutively collected breast cancer patients treated at a tertiary referral unit (Galway University Hospital) entered into a prospectively maintained database (1999–2005). Only patients with a definitive subtype were included. Multiple clinical-pathological details were selected as indicated and used for further analysis. Tumours were staged according to the International Union against Cancer’s Tumour-Node-Metastasis (TNM) classification and histologically subtyped according to WHO guidelines. A total of 337 patients had Tip60 staining results with matched clinical information (227 with a clinically defined subtype), including survival and outcome data for 334 patients. The clinicopathological characteristics corresponding to each individual with Tip60 staining data was collected and collated and are shown in Table 1.

**Table 1:** Breast Cancer Tissue Microarray: Clinicopathological Details.

Stained for Tip60 N (%)	
**Age**	
Pre/Peri-menopausal	93 (30.7%)
Post-menopausal	210 (69.3%)
Total (n=)	303
**Subtyp**e	
Luminal A	119 (35.3%)
Luminal B	28 (8.3%)
Her2 positive	16 (4.8%)
Basal	64 (19%)
Unknown	110 (32.6%)
Total (n=)	337
**Receptor Status**	
ER Positive	102 (33.3%)
ER Negative	204 (66.7%)
Total (n=)	306
PR positive	115 (38.1%)
PR Negative	187 (61.9%)
Total (n=)	302
Her2 positive	186 (81.6%)
Her2 negative	42 (18.4%)
Total (n=)	228
**Stage**	
1	65 (21.8%)
2	139 (46.6%)
3	73 (24.5%)
4	21 (7%)
Total (n=)	298
**N Score**	
0	142 (45.8%)
1	92 (29.7%)
2	55 (17.7%)
3	21 (6.8%)
Total (n=)	310
**Metastatic disease**	
No	295 (93.7%)
Yes	20 (6.3%)
Total (n=)	315

### TMA Scoring

In collaboration with clinical pathologists, a scoring system was developed based on the observed pattern of Tip60 cytoplasmic and nuclear staining in control and breast cancer specimens with staining categorised by Localisation: Cytoplasmic/Nuclear (Nuc) and Intensity: Negative, Weak, Moderate or Strong [nuclear (Nuc), cytoplasmic (Cyto), Double positive (DP; cytoplasmic and nuclear) and Double Negative (DN; no staining in both cytoplasm and nucleus), Cytoplasmic by intensity (Cyto-Weak, Cyto-Mod, Cyto-Strong)] (Fig. 2). The scoring system was utilised by two independent researchers (of which one is a practicing clinical pathologist) who independently scored the Tip60 stained TMA images in a blinded analysis. Independent analysis of the TMA scoring was performed by the study biostatisticians. TMA staining antibodies as indicated: main figure Tip60 antibody (K-17: sc-5727, Santa Cruz); Supplemental figures: Tip60 antibody PAB18305 (Abnova).

**Figure 1:**
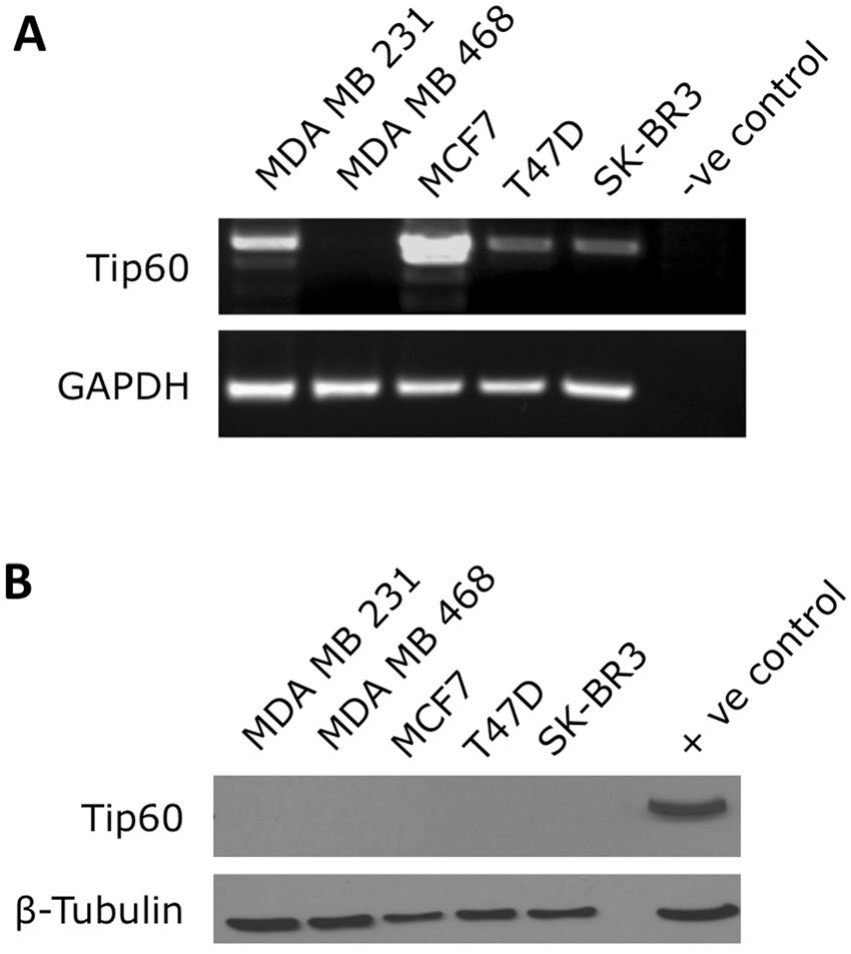
Tip60 mRNA and protein expression in breast cancer cell lines. (**A**) Tip60 expression mRNA in indicated breast cancer cell lines. Total mRNA was extracted and RT-PCR performed to create cDNA. Equal amounts of cDNA were then used in a PCR reaction to amplify a region of Tip60 and GAPDH. Control: primer only reaction.(**B**). Tip60 protein expression in indicated breast cancer cell lines: 30 μg of total protein extracted from indicated breast cancer cell lines. Positive control: human tissue extract. Antibodies used: anti-Tip60 and anti-β Tubulin.

**Figure 2:**
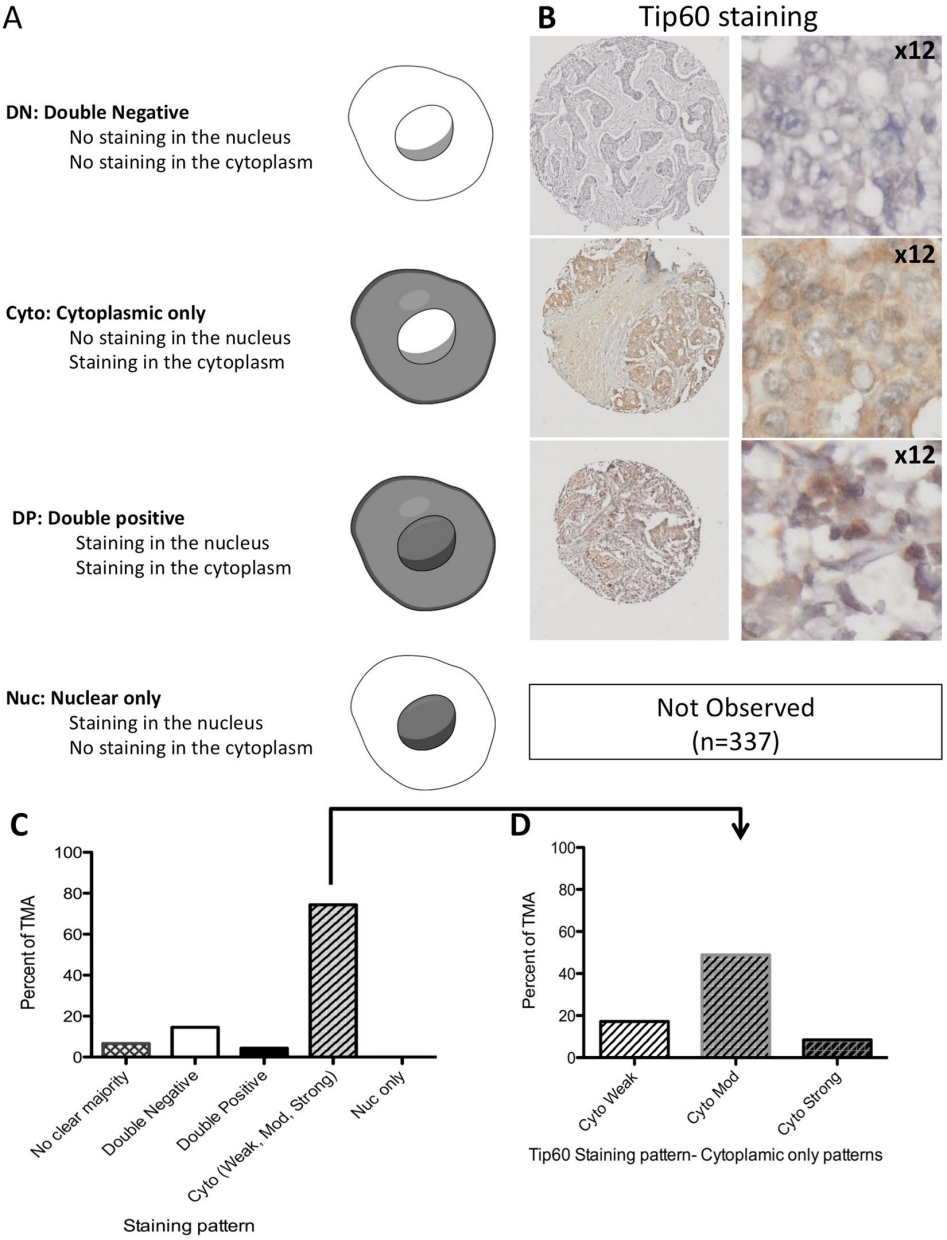
Tip60 staining in breast cancer. (**A**) Diagrammatic representation of Tip60 staining patterns. (**B**) Representative IHC images of Tip60 staining patterns. Left: Tip60 stained TMA sections. Right: 12 enlargement of section. Blue: DNA stain, Orange/Brown: Tip60 staining. (**C**) Tip60 staining patterns in TMA (percent), by indicated staining pattern. (**D**) TMA cytoplasmic only Tip60 staining patterns, by TMA percentages.

### Statistical Analysis

Consultant biostatisticians performed the statistical analysis of Tip60 TMA staining, clinicopathological and survival data for the locally generated data. Associations between categorical variables were assessed by applying Cochran-Mantel-Haenszel tests as appropriate and measured via Cramer’s V statistic and Goodman and Kruskal’s Lambda statistic. To test for differences in continuous variables by categories of staining, ANOVA or non-parametric alternative Kruskal-Wallis H tests were applied. To calculate the effect size of significant differences a series of Mann-Whitney tests were used with Bonferroni correction to control Type I error rate. Significance was reached if p ≤ 0.05. Complete-Case Cox PH regression models were used to assess the relationship between Tip60 staining and the indicated clinicopathological variables for Overall Survival response and Disease Free Survival response. Analysis was performed via R statistical programming and packages.

### Kaplan-Meier plotter analysis

The Kaplan-Meier Plotter [Breast Cancer] database (integrating clinical and gene expression data) (http://kmplot.com/analysis/)^28–31^ was used to analyze the prognostic value of Tip60 (Kat5) mRNA expression. As of March 2018 the available online database had data on 5143 breast cancer patients. Search parameters used: Gene: Kat5 (Search: Kat5, Tip60, HTATIP, cPLA2, ESA1, PLIP, HTATIP1) (Affy ID: 206689_x_at); Split patients by: auto-select best cutoff; Survival: Overall Survival (OS, n = 1402) or Relapse Free Survival (RFS n = 3951); Follow up threshold: 10 (years); Censure at threshold, selected; Use only JetSet best probe set, selected; Quality control, Remove redundant samples: checked; Array quality control: exclude biased arrays selected; Intrinsic subtype, selected as indicated.

### cBioPortal analysis

The cBioPortal database [specific TCGA Breast Cancer dataset] (http://www.cbioportal.org/)^32–36^ was used to assess the somatic mutations and copy number changes to the Kat5 gene (Fig. S10). Search parameters used: Select Studies: Breast Cancer; Studies Select all: As of March 2018 8 available studies were selected [Breast Cancer (METABRIC, Nature 2012 & Nat Commun 2016; 2509 samples), Breast Invasive Carcinoma (British Columbia, Nature 2012; 65 samples), Breast Invasive Carcinoma (Broad, Nature 2012; 103 samples), Breast Invasive Carcinoma (Sanger, Nature 2012; 100 samples), Breast Invasive Carcinoma (TCGA, Provisional; 1105 samples), Breast cancer patient xenografts (British Columbia, Nature 2014; 117 samples), Mutational profiles of metastatic breast cancer (INSERM, France, 2016; 216 samples), The Metastatic Breast Cancer Project (MBC, Provisional, October 2017; 103 samples)]; Select Data Type Priority: Mutation and CAN selected; Enter Gene Set: Kat5. Following query submission results returned and displayed were genomic alterations (including somatic mutations) and copy number variation.

### Ethical Approval

This study was conducted with ethical approvals from the Galway University Hospitals Clinical Research Ethics Committee (C.A.151 and C.A.1012), and all experiments were performed in accordance with the relevant guidelines and regulations. Use of patient material in this study was approved by the Galway University Hospitals Clinical Research Ethics Committee, meetings on the 3^rd^ of June 2008 (C.A.151) and 23^rd^ January 2014 (reference C.A.1012). The ethics committee waived the need for consent. All patients had histologically confirmed breast cancer. Relevant clinicopathological data was obtained from a prospectively maintained breast cancer database.

## Results

### Tip60 (Kat5) expression in breast cancer cell lines

To explore the cellular importance of Tip60 in breast cancer, Tip60 (Kat5) expression (both transcript and protein) in common representative breast cancer cell lines was investigated (Figs 1 and S1). Examining Kat5 mRNA transcript levels, low levels of Tip60 gene expression was seen in 4/5 cell lines (Fig. 1A). Exploring if the variable gene expression observed resulted in changes to protein expression, Tip60 protein was undetectable (under our conditions in the representative breast cancer cell lines studied) (Fig. 1B). Immunohistochemical staining of normal and breast cancer cell lines revealed barely detectable Tip60 staining (Fig. S1).

### Quantifying Tip60 localisation and intensity in breast cancer samples

According to the Human Protein Atlas the normal localization of Tip60 protein is to the nucleoplasm^37^. Employing a comprehensive scoring system Tip60 staining in a breast cancer tissue microarray (TMA) was graded, by recording a detailed characterisation of Tip60 localisation: nuclear (Nuc), cytoplasmic (Cyto), Double positive (DP; cytoplasmic and nuclear) and Double Negative (DN; no staining in both cytoplasm and nucleus) (Figs 2A,B and S2–3). This was combined with scoring of Tip60 staining intensity (weak, medium and strong) and quantification (percentage of cells) of each pattern, in each TMA biopsy.

Immunohistochemical staining of Tip60 was performed on the breast cancer TMA (n = 337; cohort clinicopathological details, Table 1). Investigating TMA samples with subtype related data (n = 227) the most common, majority staining pattern was cytoplasmic only (Cyto-only) (74.4%, n = 169), followed by DN (14.5%, n = 33) and DP (4.4%, n = 10), with 6.6% (n = 15) showing no clear majority (2 patterns having equal proportions). Interestingly, no Nuc only (0%) staining (the “normal” Tip60 localisation) was observed (Fig. 2C). Further subdividing the Cyto-only staining pattern by intensity (Weak, Moderate, Strong), the most prevalent pattern was Cyto-Mod (48.9%, n = 111), followed by Cyto-Weak (17.2%, n = 39) and Cyto-Strong (8.4%, n = 19) (Figs 2D and S4A).

### Analyzing Tip60 TMA staining patterns

**Figure 3:**
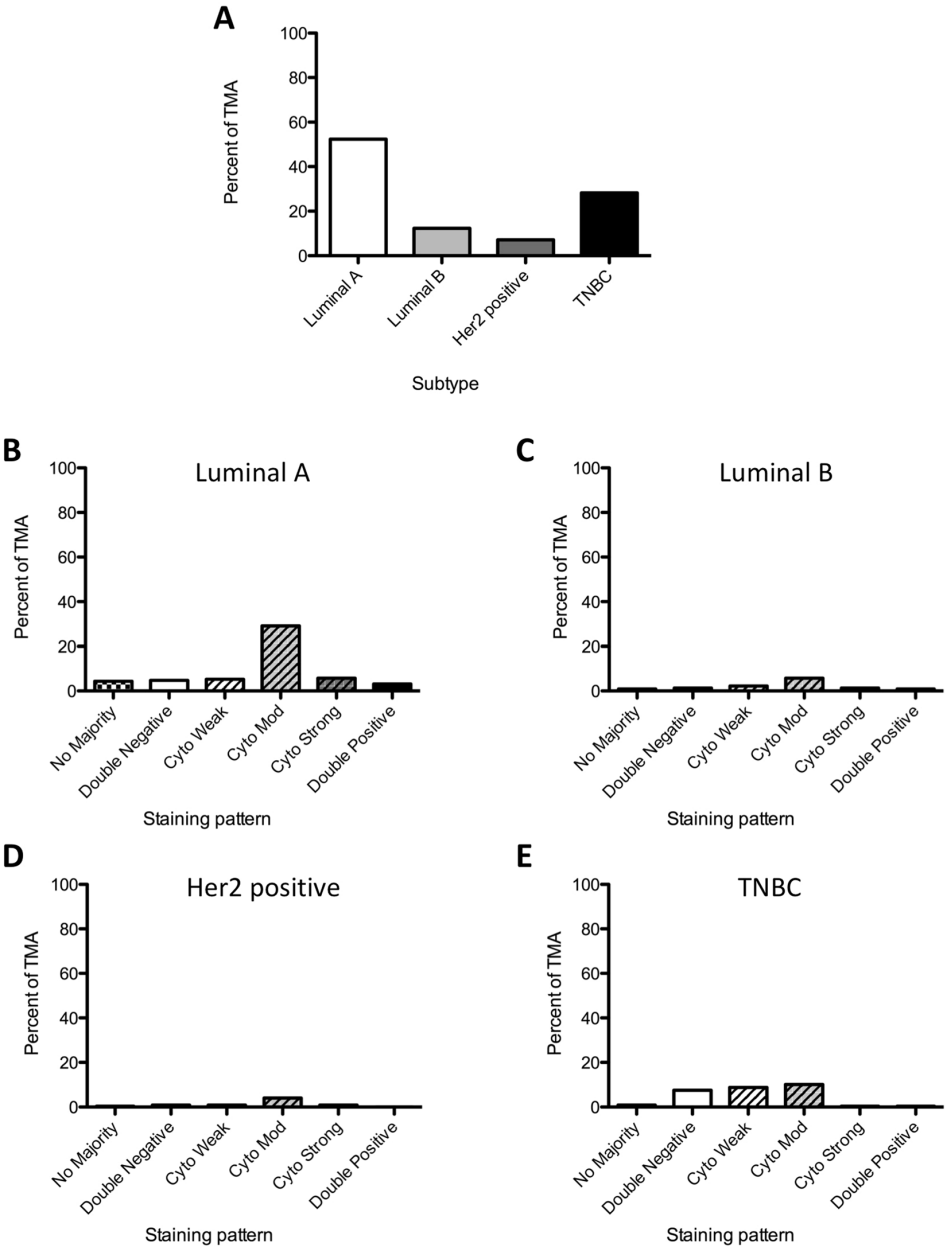
Association of Tip60 intensity and clinicopathological details. (**A**) TMA by breast cancer subtype (percent TMA)(n = 337). (**B**–**E**) TMA by breast subtype (indicated) and Tip60 staining pattern (percent of TMA).

Analyzing the TMA by subtype, the TMA loosely follows the subtype proportions observed clinically: Luminal A (52.4%, n = 119), Luminal B (12.3%, n = 28), Her2 positive (7%, n = 16), with an overrepresentation of the Basal/Triple Negative subtype (28.2%, n = 64) (Fig. 3A). Exploring the staining pattern by subtype Cyto-Mod is the predominant pattern in Luminal A (29.1%), Luminal B (5.7%) and Her2 positive (4%) (Fig. 3B–D, Table 2). In the Luminal A cohort the Cyto-Mod pattern accounts for 55.5% (n = 66) of patients (Fig. S4B). The TNBC subtype displayed a clearly different Tip60 staining pattern distribution, with roughly equal proportions observed in the DN, Cyto-Weak and Cyto-Mod patterns (7.5%, 8.8% and 10.1% respectively)(Fig. 3E, Table 2).

**Table 2:** Quantification of Tip60 staining pattern in breast cancer TMA.

Subtype	Tip60 staining pattern % TMA (n)					
	No Majority	DN	Cyto-Weak	Cyto-Mod	Cyto-Strong	DP
Luminal A	4.4% (n = 10)	4.8% (n = 11)	5.3% (n = 12)	**29.1% (n** = **66)**	5.7% (n = 13)	3.1% (n = 7)
Luminal B	0.9% (n = 2)	1.3% (n = 3)	2.2% (n = 5)	**5.7% (n** = **13)**	1.3% (n = 3)	0.9% (n = 2)
Her2 positive	0.4% (n = 1)	0.9% (n = 2)	0.9% (n = 2)	**4% (n** = **9)**	0.9% (n = 2)	0% (n = 0)
TNBC	0.9% (n = 2)	7.5% (n = 19)	8.8% (n = 20)	**10.1% (n** = **23)**	0.4% (n = 1)	0.4% (n = 1)

**Figure 4:**
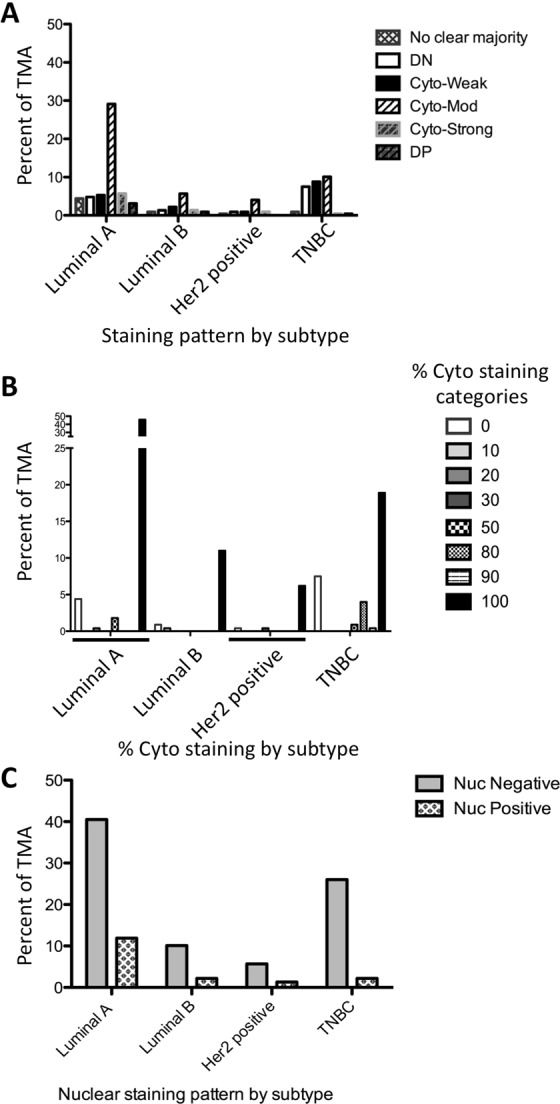
Breast cancer subtype TMA Tip60 staining patterns. (**A**) grouped Tip60 staining pattern by subtype. (**B**) Percent of cytoplasmic only Tip60 staining, by subtype. (**C**) Nuclear Tip60 staining positivity by subtype.

Testing the association between Tip60 staining intensity categories and breast cancer subtype, there was significant evidence to suggest a general association in the population (p = 0.0046) (n = 227) (Fig. 4A). Further analysis of the total-Cyto-only (cyto-only staining in all patterns) staining in each subtype, by percent of cells stained, a significant association between total-Cyto-only percent (0, 10, 20, 30, 50, 80, 90 and 100%) and breast cancer subtype (p = 0.0055) was seen (Fig. 4B). Testing the association between nuclear staining (+/−) and breast cancer subtype, no significant association was observed (p = 0.0964) (Fig. 4C).

### Evaluating Tip60 staining patterns and clinicopathological variables

**Figure 5:**
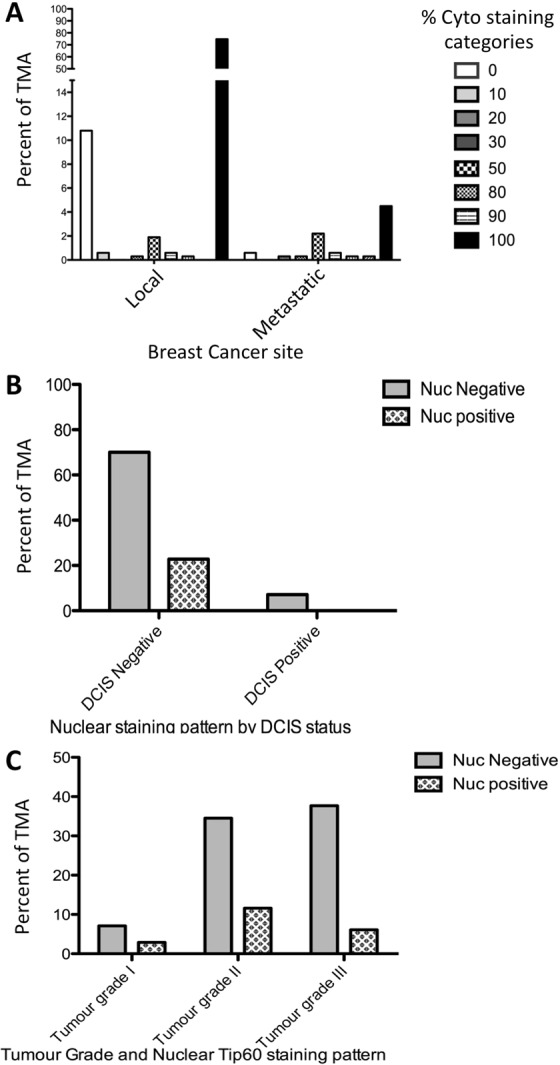
Key breast cancer clinicopathological variables associated with TMA Tip60 staining patterns. (**A**) Percent of cytoplasmic only Tip60 staining, by cancer site. (**B**) Nuclear Tip60 staining positivity by type. Ductal Carcinoma *In Situ* considered non-invasive/pre-invasive. (**C**) Nuclear Tip60 staining positivity by indicated tumour grade.

Investigating the association between total-Cyto only staining percentage and location of breast cancer (local or metastatic) (n = 315) a significant association was found (p = 0.0002) (Fig. 5A). A significant association with Nuc Tip60 staining (p = 0.0057) was observed when the cohort (n = 337) was categorised as either the early or precancerous/non-invasive (intra-ductal) Ductal Carcinoma *In Situ* (DCIS) or not (Fig. 5B). While no significant association was found between Tip60 majority staining pattern and Tumour grade (p = 0.4511), it was interesting to note that in Grade II and III tumours the majority of staining observed had a cytoplasmic component (Figs S2, S3, S5A). Investigating any association between Tumour grade and Tip60 Nuc staining categories (+/−) in the population, a significant association (p = 0.0334) was found (Fig. 5C). Testing the association between total Cyto staining percentage and UICC stage, a strong correlation (p = 0.0067) was observed (Fig. S5B).

**Figure 6:**
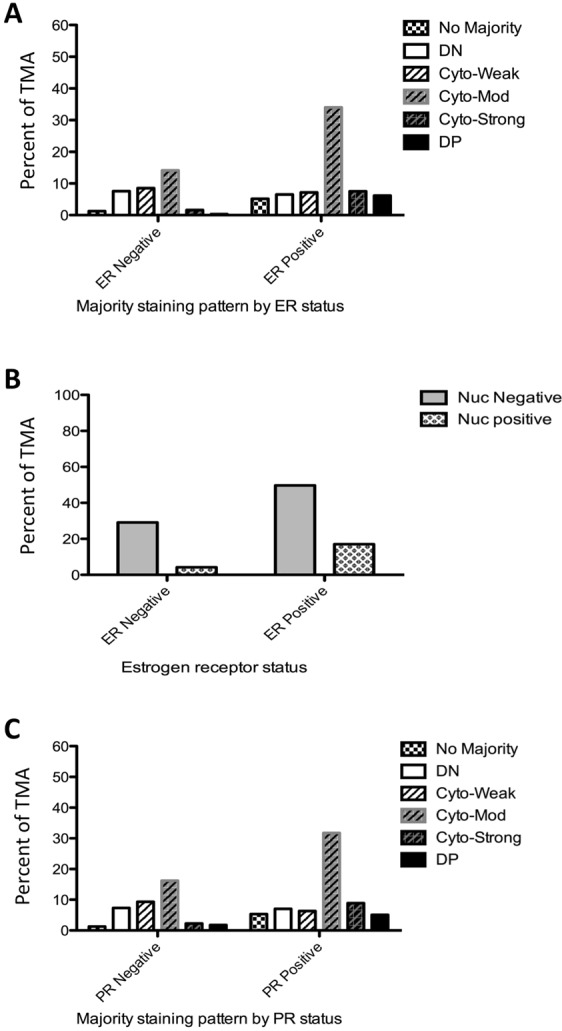
Breast cancer receptor status is associated with TMA Tip60 staining patterns. (**A**) ER staining positivity by Tip60 staining patterns. (**B**) Nuclear Tip60 staining positivity by ER status. (**C**) PR staining positivity by Tip60 staining patterns.

Investigating the association between ER (Estrogen Receptor) status (n = 306) and Tip60 staining patterns, there was evidence to suggest a significant association with the staining intensity of Tip60 in the population (p ≈ 0.000) (Fig. 6A). A significant association is also observed when the Cyto staining intensity categories are grouped (p = 0.0008) (Fig. S6A). Exploring ER status by individual categories of Cyto only staining percentage, no significant association (p = 0.5198) was found (Fig. S6B). Exploring the association between Tip60 Nuc staining categories (+/−) and ER status in the population, there was evidence to suggest a significant association (p = 0.0103) (Fig. 6B). Investigating the association with PR (Progesterone Receptor) status (n = 302), there was a significant association (p = 0.0017) between the majority staining patterns and PR status in the population (Fig. 6C). However, no significant association in the population (p = 0.0621) was found when the Cyto staining intensities categories were grouped (Fig. S7A). Exploring individual categories of Cyto only staining by percentage stained and PR status, no significant association was found (p = 0.6207, data not shown). Investigating any association between PR status and Tip60 Nuc staining categories (+/−) in the population, there was no evidence to suggest any significant association in the population (p = 0.1714, Fig. S7B).

No significant association was found between any Tip60 staining patterns (majority staining pattern, p values shown) and: Her2 (p = 0.9274), Tumour size (p = 0.1123), N-Score (p = 0.7738), Nottingham Prognostic index (NPI) (p = 0.6026) or Age (p = 0.1735) (Data not shown). Investigating the association between nuclear staining pattern and Menopausal status (n = 303) no evidence of an association testing at significance level α = 0.05 was found (p = 0.0522, Fig. S8A).

### Tip60 staining patterns and patient survival (OS, RFS)

As expected, using Univariate complete-case Cox proportional hazard (CPH) regression models for OS, our cohort conformed to previous studies demonstrating that there is evidence that Subtype (n = 227, p = 0.0023), Tumour Size (n = 316, p = 7.3e−05), Stage (n = 296, p = 1.6e−09) and Age (n = 334, p = 8.2e−08) have a significant effect on survival outcomes (Fig. S9A–D). Investigating Tip60 majority staining patterns (DN, DP and Cyto-only)(n = 334) no significant association with OS (p = 0.25) was seen. Investigating Tip60 majority staining patterns (n = 313) and RFS, again no significant association (p = 0.43) was observed.

### Modeling Tip60 staining patterns and survival

CPH modeling which uses and analyzes only data from patients with complete covariate data, but can be considered inefficient as not all data in the cohort is utilized. Therefore this approach was combined with a more efficient imputed-data CPH modeling, which incorporated data from all cohort patients (featuring a predictive model based on observed data to estimate missing covariate values). Using complete-case CPH modeling (n = 185) of OS, the variables Cyto-Only percentage and Stage explain the variability in OS (Table 3, Fig. S10A). Fitting this model to all complete-cases which contain those two key variables (n = 296) Stage remained significant, but not Cyto-Only percentage. Using imputed-data CPH modeling for OS three clinicopathological variables (Subtype, Stage and Age)(n = 334) were found to explain the variability in OS (Table 4, Fig. S10B).

**Table 3:** CPH modeling of Tip60 staining pattern and Clinicopathological data.

Tip60 staining pattern	Overall Survival Time HR (95% CI)
Cyto-Only percentage	0.991^***^ (0.985, 0.997)
Stage II	1.859 (0.701, 4.932)
Stage III	5.973^***^ (2.258, 15.797)
Stage IV	12.589^***^ (3.498, 40.143)
n = 185	

*p < 0.1; **p < 0.05; ***p < 0.01.

**Table 4:** CPH modeling of Tip60 staining pattern and OS.

Clinicopathological data	Overall Survival Time HR (95% CI)
Luminal B	1.456 (0.821, 2.579)
Her2 positive	1.487 (0.857, 2.582)
Basal	4.454^***^ (2.856, 6.945)
Stage II	1.364 (0.762, 2.440)
Stage III	3.470^***^ (1.894, 6.357)
Stage IV	7.611^***^ (3.643, 15.940)
Age	1.038^***^ (1.024, 1.053)
n = 334	

*p < 0.1; **p < 0.05; ***p < 0.01.

Considering complete-case CPH models for DFS (n = 174), the model with the lowest Akaike information criterion (AIC; an estimator of the relative quality of statistical models for a given set of data) contained seven variables (Cyto-Only percentage, Total Cyto percentage, Nuc, Subtype, NPI, Stage, and Menopause status), which together explain the variability in DFS outcome (Table 5, Fig. S10C). Applying a backward stepwise variable selection routine gives the model (n = 174) with four variables Cyto-Only percentage, Total Cyto percentage, Subtype and Stage. When fitting the same model specification to all 193 complete-case individuals (with data for all four variables), the variables Subtype and Stage remain significant, however Cyto-Only percentage, Total Cyto percentage are not.

**Table 5:** CPH modeling of Tip60 staining pattern and DFS.

Clinicopathological data	Overall Survival Time HR (95% CI)
Cyto-Only percentage	**0.966** ^***^ **(0.947, 0.985)**
Total Cyto percentage	**1.033** ^***^ **(1.011, 1.054)**
Nuc	**0.211** ^***^ **(0.052, 0.848)**
Luminal B	0.488 (0.186, 1.284)
Her2 positive	0.839 (0.330, 2.135)
Basal	**1.912** ^***^ **(1.038, 3.522)**
NPI- 2	0.676 (0.079, 5.794)
NPI- 3	0.702 (0.081, 6.107)
NPI- 4	1.516 (0.159, 14.442)
Tumour size (mm)	0.992 (0.974, 1.010)
Stage II	1.367 (0.543, 3.442)
Stage III	2.737 (0.809, 9.262)
Age	0.994 (0.961, 1.028)
Menopause (post)	1.560 (0.635, 3.832)
n = 174	

*p < 0.1; **p < 0.05; ***p < 0.01.

### Tip60 (Kat5) mRNA expression and survival/relapse rates in an independent cohort

**Figure 7:**
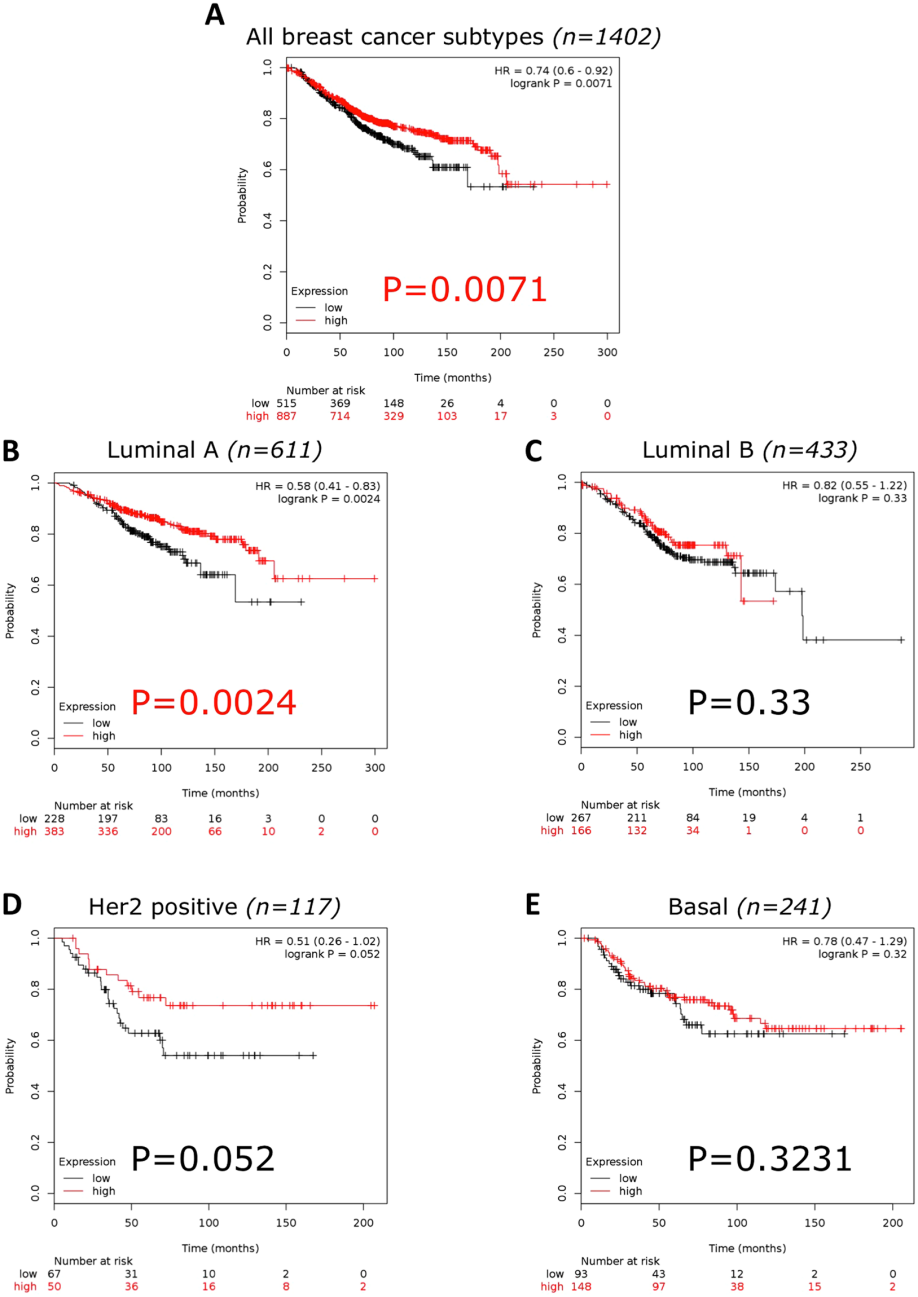
Kaplan Meier of Kat5 (Tip60) mRNA expression and Overall Survival in breast cancer. (**A**) All breast cancer subtypes (n = 1402 and p = 0.0071). (**B**) Luminal A (n = 611 and p = 0.0024). (**C**) Luminal B (n = 433 and p = 0.33). (**D**) Her2 receptor positive (n = 117 and p = 0.052). (**E**) Basal subtype (n = 241 and p = 0.3231). Generated using Kaplan-Meier plotter^29^. Microarray data, using only JetSet best probe set.

To determine if evaluating Tip60 (Kat5) mRNA expression in tumours could be clinically relevant, a large online data set was utilised^29^. Investigating the difference in Kat5 expression between normal (n = 76) and breast cancer (n = 6547) samples, a significant difference was found (p = 0.0000000332) [median 453 (range 84–1205) v 605 (range 11–2125)]^29^. Exploring the relationship between Kat5 expression and overall survival (OS) in breast cancer, low Kat5 expression significantly correlated (p = 0.0071) with reduced OS (n = 1402) (Fig. 7A). Examining individual breast cancer subtypes a significant correlation (p = 0.0024) with low Kat 5 expression and decreased OS in Luminal A (n = 611) was found. No significant association was observed in Luminal B (n = 433; p = 0.33), Her2 positive (n = 117; p = 0.052) or Basal subtypes (n = 241; p = 0.3231) (Fig. 7B–E).

**Figure 8:**
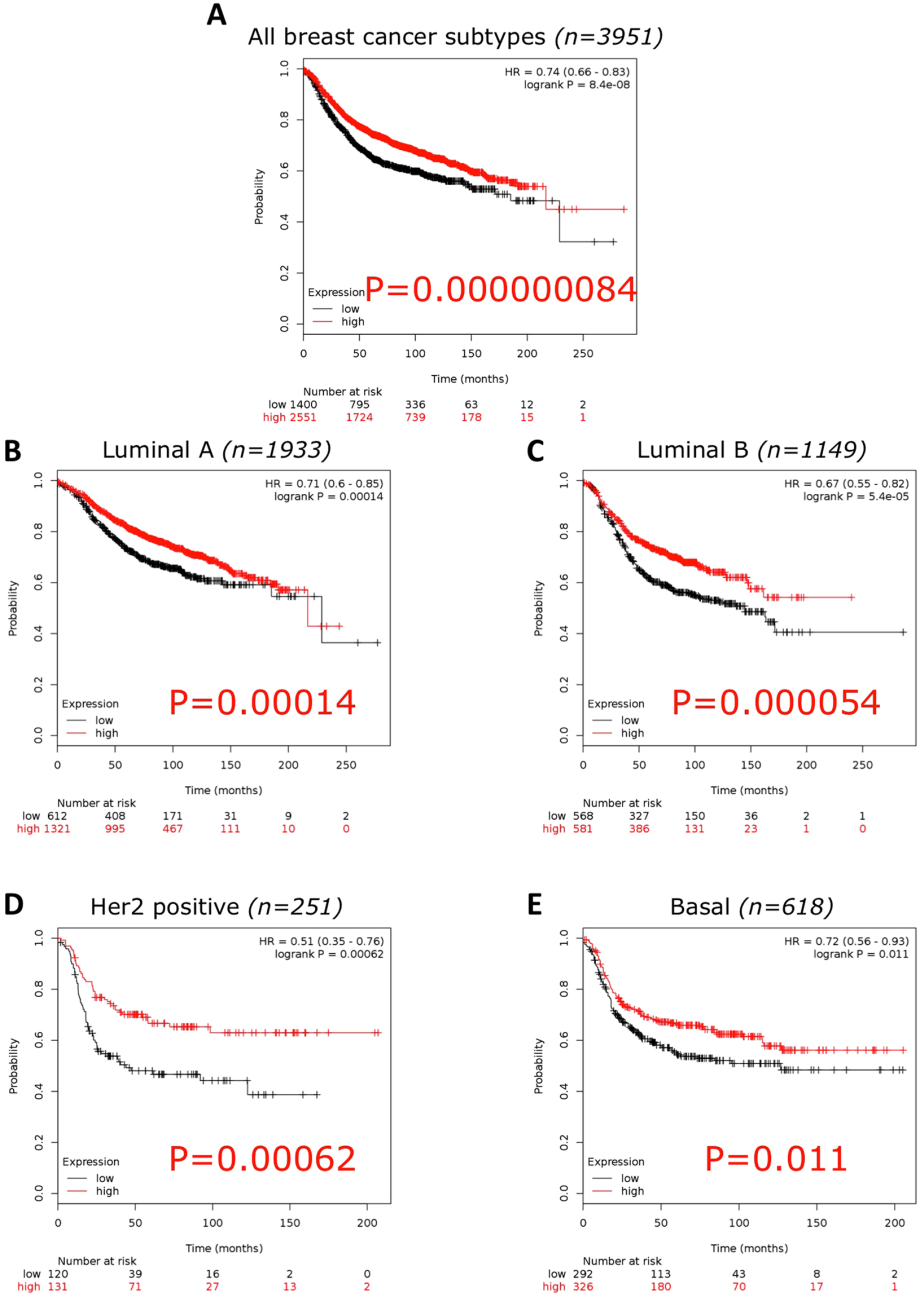
Kaplan Meier of Kat5 (Tip60) mRNA expression and Relapse Free Survival in breast cancer. (**A**) all breast cancer subtypes (n = 3951 and p = 0.000000084). (**B**) Luminal A (n = 1933 and p = 0.00014). (**C**) Luminal B (n = 1149 and p = 0.000054). (**D**) Her2 receptor positive (n = 251 and p = 0.00062). (**E**) Basal subtype (n = 618 and p = 0.011). Generated using Kaplan-Meier plotter^29^. Microarray data, using only JetSet best probe set.

Examining the correlation between low Kat5 expression and Relapse Free Survival (RFS) in breast cancer a significant correlation (p = 0.000000084) was seen (n = 3951) (Fig. 8A). Examining individual breast cancer subtypes, low Kat5 expression significantly correlated with decreased RFS in all subtypes: Luminal A (n = 1933; p = 0.00014), Luminal B (n = 1149; p = 0.000054), Her2 positive (n = 251; p = 0.00062) and Basal (n = 618; p = 0.011) (Fig. 8B–E).

Using data from 8 independent breast cancer cohorts (cBioPortal^33^, n = 3627) the overall rate of Kat5 gene amplification was observed to be 1.6% (n = 58). A deletion of Kat5 was found in only 0.11% (n = 4) of samples (Fig. S11A). Investigating somatic Tip60 mutations, a rate of only 0.1% was observed (n = 3; 2 missense, 1 truncating) (Fig. S11B).

## Discussion

Tip60 is a key member of the MYST family of acetyltransferases, a class of enzymes that are becoming increasingly interesting as targets of drug development^3,38^. The effective deployment of KAT inhibitors will require patient stratification, allowing tumours with the greatest sensitivity to be selected, to produce the most efficient killing of tumourigenic cells. Interestingly, Tip60 protein expression was not seen *in vitro*, however as Tip60 is an essential protein^13,14^ it is highly likely that the expression was below the detection threshold of our assay, which was confirmed as very low levels of Tip60 protein were detected by IHC staining. We then evaluated the ability to score and quantify Tip60 levels (protein and mRNA) in breast cancer *in vivo*, and correlated this with key clinicopathological criteria.

We graded breast cancer tumour samples in a detailed and comprehensive way, according to Tip60 staining intensity and cellular localisation. We found that the most common Tip60 staining pattern was cytoplasmic only (>74%), and within this the Moderate intensity was the predominant staining pattern (>48%). Importantly we did not see any normal (nuclear only) staining. This further supports the idea that Tip60 haploinsufficiency in breast cancer results in Tip60 functioning as an oncogene^12^. Importantly, overall we correlated low Tip60 expression with reduced survival in breast cancer patients, extending previous work^39^, and specifically in the Luminal A subtype. Importantly, low Tip60 expression led to increased incidences of tumour recurrence and in all individual breast cancer subtypes. Advancing the idea that the loss of Tip60 expression is required for cancer progression, we observed an association of mislocalised Tip60 staining (cytoplasmic) with increasing tumour stage. While this supports a mechanism for tumour progression requiring Tip60 down regulation, we did observe a very low rate of Kat5 gene amplification (1.6%). However the cellular and molecular consequences of Tip60 overexpression remain unknown. The role of the cytoplasmic mislocalised Tip60 in breast cancer is a key unanswered question, as in the nucleus it functions primarily as a transcription factor and DSB regulator. However, in lung cancer cells cytoplasmic Tip60 has been shown to be regulated by HDAC3, which prevents Tip60-dependent apoptosis^40^. Further work is needed to determine what are the functional consequences of cytoplasmic Tip60 in breast cancer, and if any functions are dependent on the amount (intensity) of Tip60 in the cytoplasm. This may also reveal potential Tip60-dependent oncogenic mechanisms that may have therapeutic implications.

In our breast cancer cohort we found the predominant staining pattern of moderate cytoplasmic Tip60 staining was displayed by >55% of Luminal A patients. Additionally, the Luminal A cohort displayed a significant number of cells with nuclear staining, indicating that in these tumours it is likely that some of the normal functions of Tip60 are retained, including pro-apoptotic functions, which may relate to the improved outcome in Luminal A patients. Overall, we found a correlation between low Tip60 staining and DCIS or tumour grade, supporting previous work^12^. Interestingly, in TNBC tumours we observed heterogeneous Tip60 staining patterns, however larger numbers would be needed to confirm if this is a relevant biomarker. These mixed Tip60 patterns seen may be a key marker that demonstrates that TNBC are heterogeneous tumours and may support a mechanism for therapy resistance in TNBC tumours, or highlight a process of progression of these tumours to a more aggressive phenotype. Transcriptomic profiling of Tip60 double negative staining cells may reveal key mechanisms that allow cancer cells to survive with minimal levels of Tip60, and emphasize key protective pathways in these cells. The effects of re-expressing Tip60 in breast cancer cells with varying levels of endogenous Tip60 expression would allow evaluation if the down-regulation/mislocalisation of Tip60 is part of an anti-apoptotic mechanism for tumour progression in breast cancer and if it is a cause or consequence of progression. Further work investigating the effects of chemotherapeutics on Tip60 expression would be informative, and based on our results here we propose that metastatic or refractory tumours are likely to be Tip60 low/negative. Further work in a targeted cohort of patients (with primary and metastatic/recurrent tumours) would be needed to confirm if low/negative Tip60 is a prognostic marker of poor outcome.

Here we demonstrated a correlation between Tip60 staining and ER or PR status, particularly nuclear Tip60 staining. This supports a role for Tip60 in ER positive breast cancer, where Tip60 was identified as a dual-function co-regulator of ERβ1, modulating ERβ1 target gene expression by suppressing the activity of ERβ1 at estrogen-response elements^41^. This is further supported by the Tip60 dependent estrogen-induced transcription of a set of estrogen receptor alpha (ERα) target genes. This Tip60 and ERα interaction is required for estrogen-induced transcription^42^. Here, using a cohort which was almost 70 times larger than the single previous report^12^, we found the rate of Kat5 allelic loss is only 0.11%. Our figure may represent a true reflection of the incidence of Kat5 allele loss, suggesting that Tip60 down-regulation *in vivo* is caused by another mechanism that requires further work to elucidate.

Interestingly, while Tip60 protein was barely detectable in breast cell lines the transcript levels were detectable in almost all lines (semi-quantitative RT-PCR assay), which supports the microarray results in tumours (Figs 7–8). Together this suggests that regulation of Tip60 may occur at either the post-transcriptional level, though active degradation of the Tip60 protein, or as previously suggested mediated by haploinsufficiency^12^. We found that Tip60 protein scoring is a biomarker to stratify breast cancer patients into specific cohorts, and low Tip60 expression (determined by microarray) is a significant risk factor indicating a higher chance of disease recurrence. However, much remains to be uncovered about the molecular role of Tip60 in breast cancer initiation and progression. The essential role of Tip60 in the DNA double strand break response, and in transcriptional regulation of many key genes, makes it an attractive marker indicating higher risk breast cancer and may provide a useful drug target for the treatment of these high risk patients.

## Supplementary information

Supplementary Figures S1–11

## Data Availability

The datasets generated for this study are available from the corresponding author on reasonable request.
