# Supplementary material for: Quantifying Tip60 (Kat5) stratifies breast cancer
Source: Sci Rep. 2019 Mar 7;9:3819. doi: 10.1038/s41598-019-40221-5 (PMC6405843; doi:10.1038/s41598-019-40221-5)
Supplement: Supplementary file 1 — Supplementary Figures S1–11 [file 41598_2019_40221_MOESM1_ESM.pdf]

Quantifying Tip60 (Kat5) stratifies breast cancer.

Authors: McGuire A[1], Casey MC[1], Shalaby A[2], Kalinina O[3], Curran C[1], Webber M[2], Callagy G[2], Holian E[3], Bourke E[2], Kerin MJ[1], Brown JA[1 ϕ*]

Supplemental figures and legends.

Supplemental Figure 1. A. Tip60 IHC staining in Control cell line (MCF10A) and indicated Breast Cancer Cell lines. Left, full size image; Right, 5x magnification. Blue: DNA counter stain, Orange/Brown: Tip60 staining. **B.** IHC Negative control. Staining performed using Tip60 antibody PAB18305 (Abnova).

Supplemental Figure 2. Additional examples of Tip60 IHC staining in breast tumour samples (Supplementary to Figure 2B). Image Pairs: Top image: full stained TMA. Bottom image: 12x magnification of tumour. Blue: DNA stain, Orange/Brown: Tip60 staining (Tip60 antibody K-17: sc-5727, Santa Cruz).

Supplemental Figure 3. Tip60 staining of invasive breast cancer tumour with second Tip60 antibody (Abnova). Blue: DNA stain, Orange/Brown: Tip60 staining. Staining performed using Tip60 antibody PAB18305 (Abnova).

Supplemental Figure 4. A. Tip60 staining patterns observed in TMA (percent). **B.** Tip60 staining patterns by percent of Luminal A cohort.

Supplemental Figure 5. A. Tip60 staining patterns observed, by tumour grade. **B.** Tip60 staining patterns observed, by Union for International Cancer Control (UICC) tumour stage.

Supplemental Figure 6. A. Tip60 staining patterns observed by ER positivity. **B.** Percent of cytoplasmic only Tip60 staining, ER positivity.

Supplemental Figure 7. A. Tip60 staining patterns observed by PR positivity. **B.** Nuclear Tip60 staining positivity by PR status.

Supplemental Figure 8. A. Nuclear Tip60 staining positivity by menopausal status.

Supplemental Figure 9. A. Univariate complete-case Cox proportional hazard (CPH) regression modeling association with: **A.** Subtype (n=227). **B.** Tumour size. **C.** Stage (n=296). **D.** Age.

Supplemental Figure 10. A. Complete-case CPH modeling (n=185) of OS and Cyto-Only percentage. **B.** Imputed-data CPH modeling for OS using Subtype, Stage and Age (n=334). **C.** Complete-case CPH modeling for DFS (n=174) using Cyto-Only percentage, Total Cyto percentage, Nuc, Subtype, NPI, Stage, and Menopause status explain variability in DFS outcome.

Supplemental Figure 11. A. Allelic changes observed (change, % and n indicated) in Kat5 gene in breast cancer cases. Using cBioPortal with individual Breast cancer studies indicated. **B.** Tip60 mutations observed in breast cancer, in cBioPortal.

S1

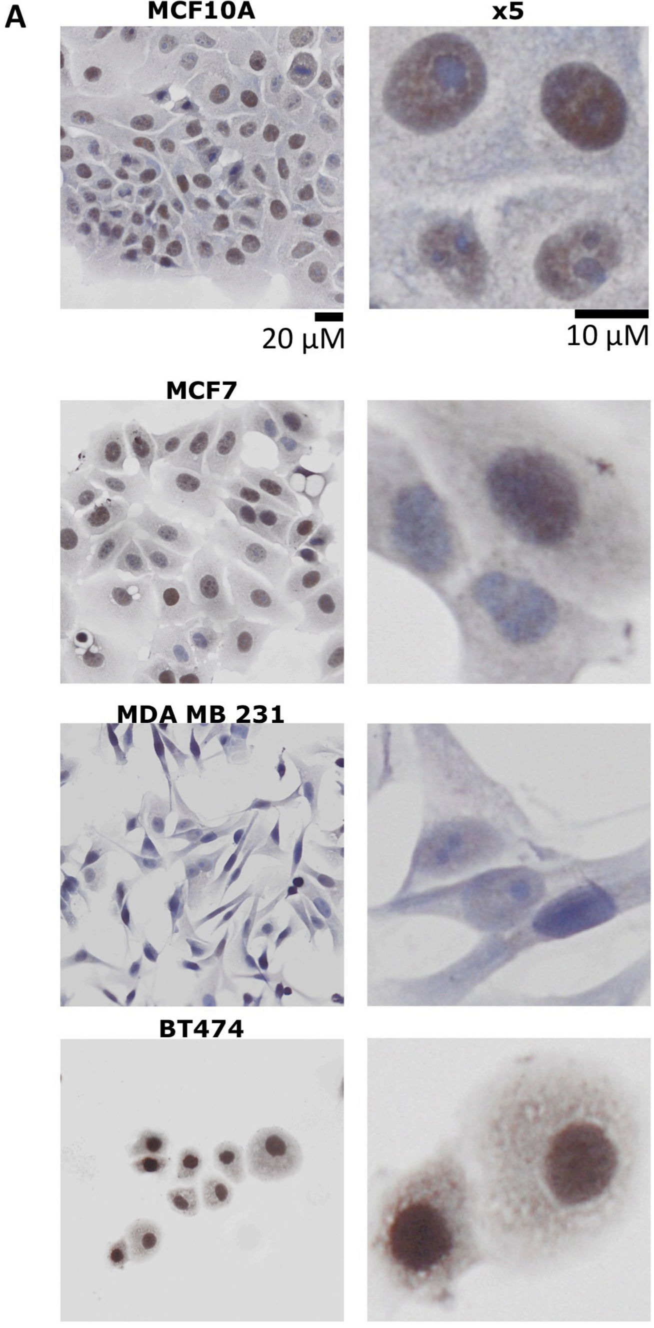
A
MCF10A
x5
20 μM
10 μM
MCF7
MDA MB 231
BT474

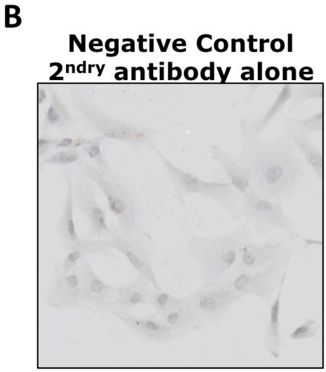
B
Negative Control
2ndry antibody alone

S2

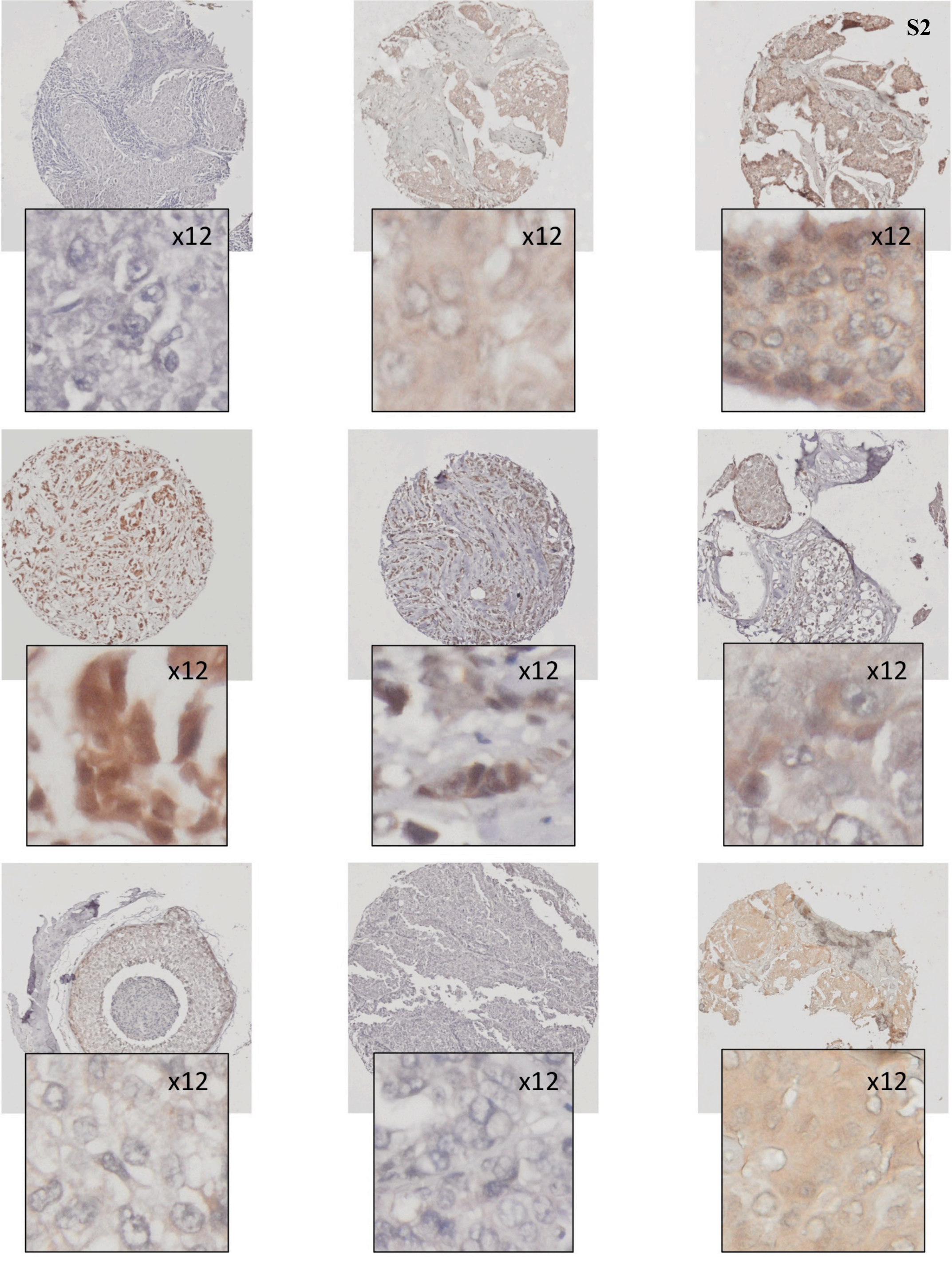

S3

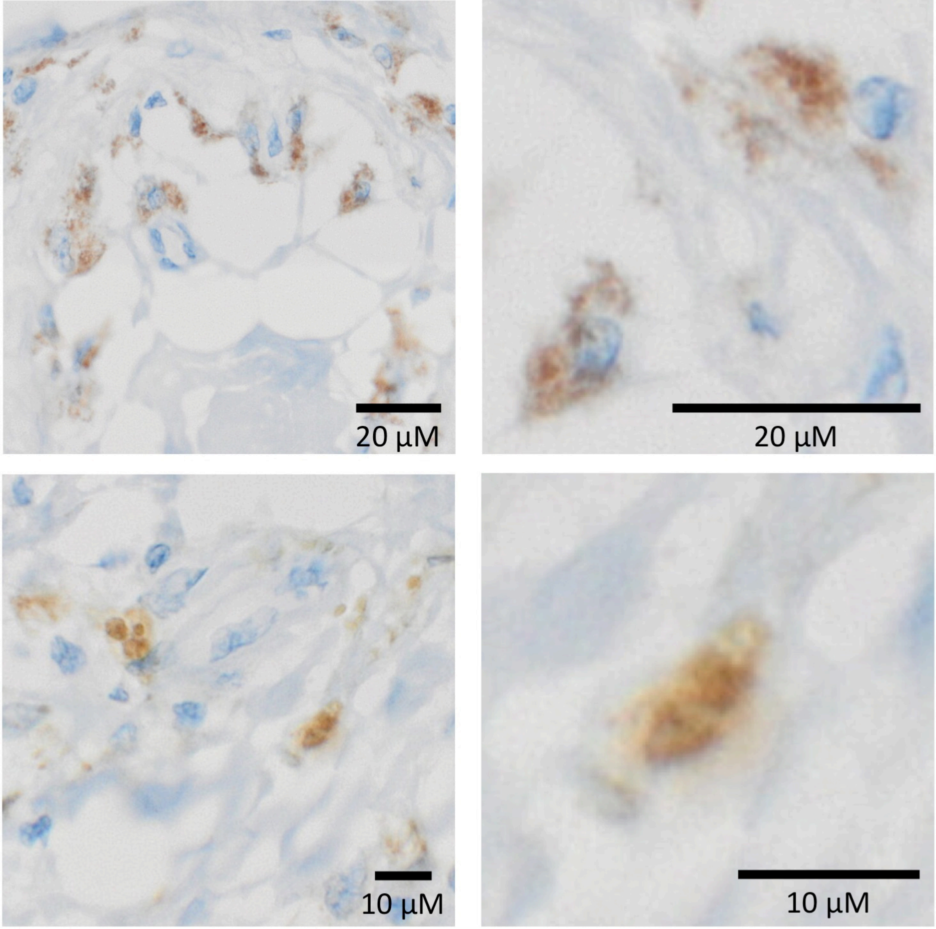

S4

A

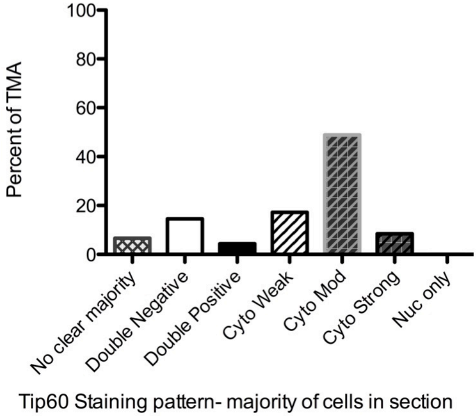

100
80
60
40
20
0
Percent of TMA
No clear majority
Double Negative
Double Positive
Cyto Weak
Cyto Mod
Cyto Strong
Nuc only
Tip60 Staining pattern- majority of cells in section

B

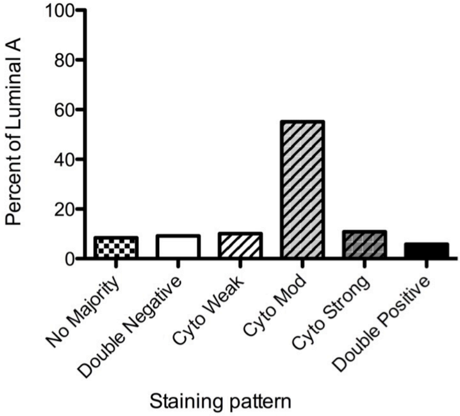

100
80
60
40
20
0
Percent of Luminal A
No Majority
Double Negative
Cyto Weak
Cyto Mod
Cyto Strong
Double Positive
Staining pattern

A

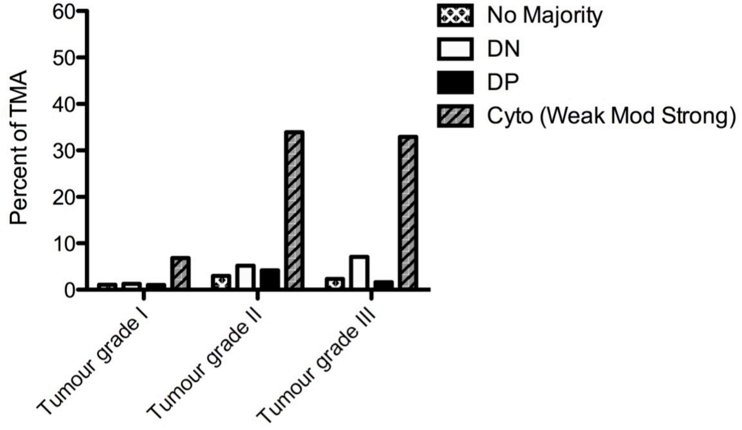

Majority staining pattern by Tumour Grade

B

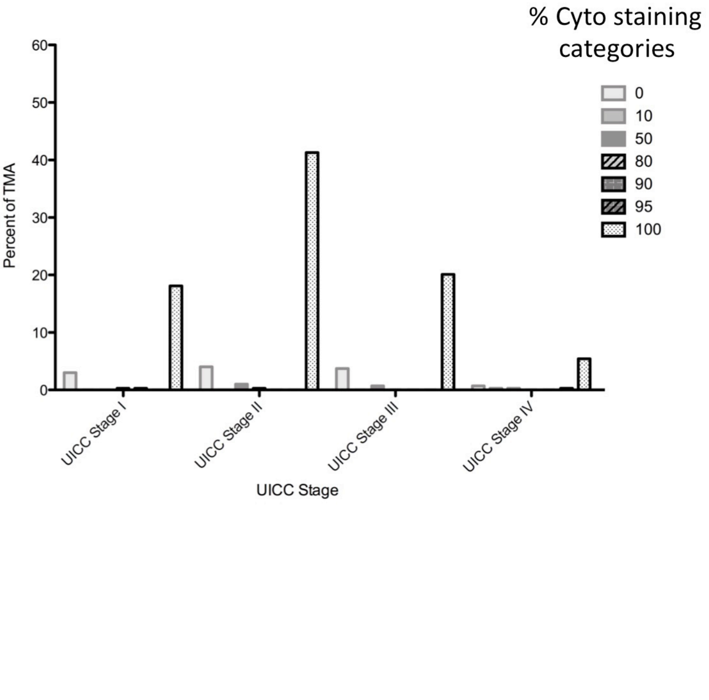

A

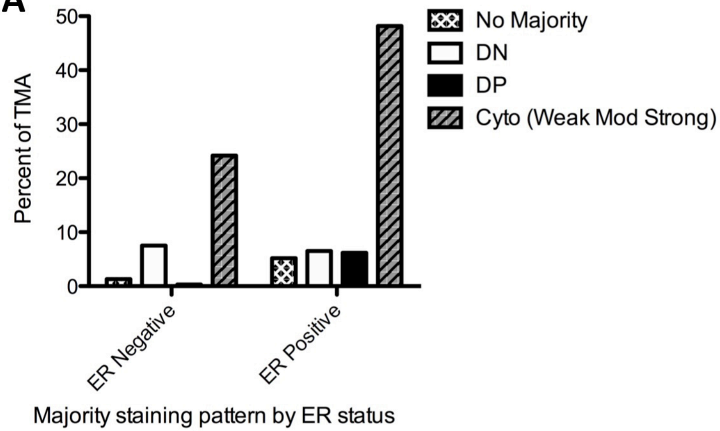
50
40
30
20
10
0
Percent of TMA
No Majority
DN
DP
Cyto (Weak Mod Strong)
ER Negative
ER Positive
Majority staining pattern by ER status

B

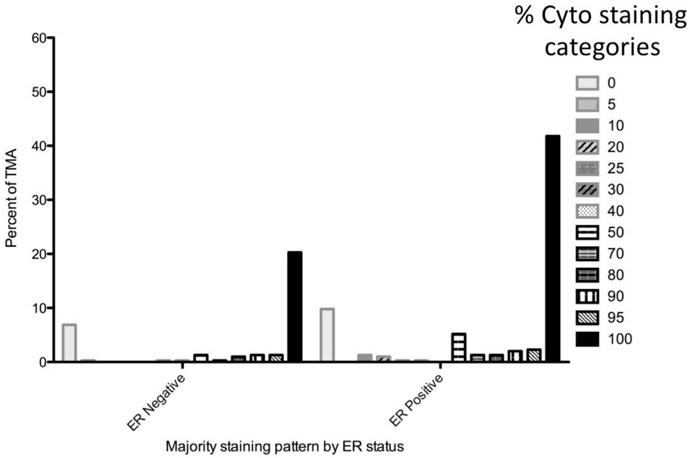
% Cyto staining categories
60
50
40
30
20
10
0
Percent of TMA
0
5
10
20
25
30
40
50
70
80
90
95
100
ER Negative
ER Positive
Majority staining pattern by ER status

S7

A

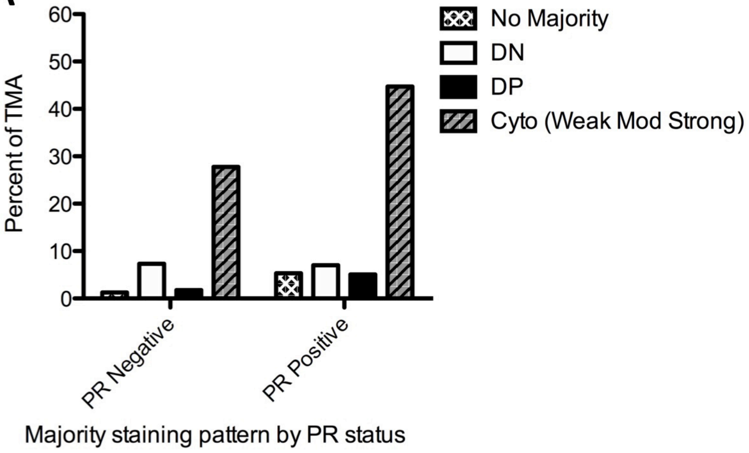

No Majority
DN
DP
Cyto (Weak Mod Strong)
Percent of TMA
0
10
20
30
40
50
60
PR Negative
PR Positive
Majority staining pattern by PR status

B

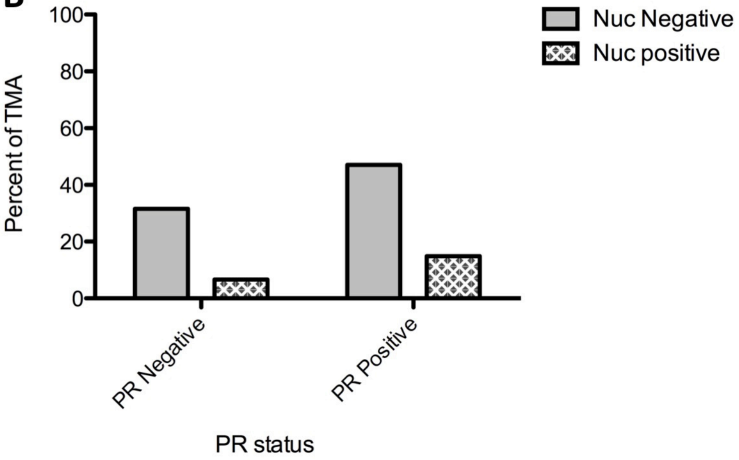

Nuc Negative
Nuc positive
Percent of TMA
0
20
40
60
80
100
PR Negative
PR Positive
PR status

A

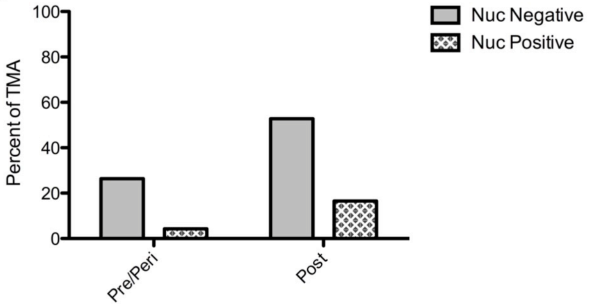

Nuclear staining pattern by menopausal status

A

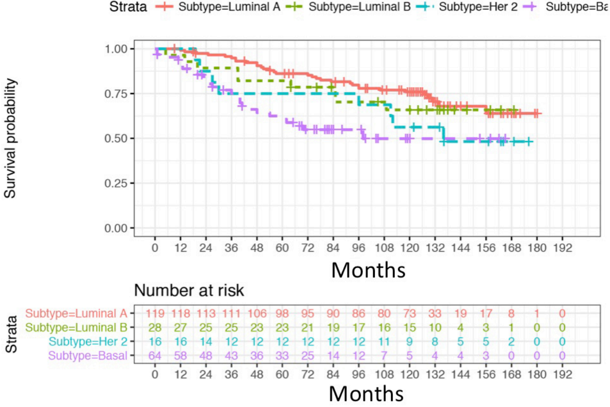

B

	Overall Survival Time
	HR(95 perc CI)
Tumour.Size	1.020***
	(1.011, 1.029)
Observations	316
Note:	*p<0.1; **p<0.05; ***p<0.01

C

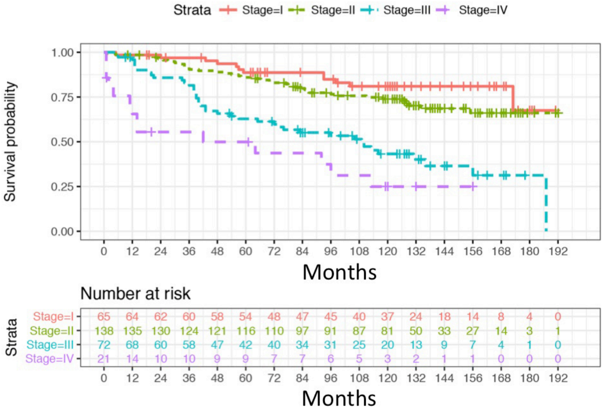

D

	Overall Survival Time
	HR(95 perc CI)
Age	1.039***
	(1.025, 1.054)
Observations	334
Note:	*p<0.1; **p<0.05; ***p<0.01

S10

A

Variable		N	Hazard ratio	p
**CytoOnly.Pct**		185	0.99 (0.99, 1.00)	0.005
**Stage**	I	41	Reference	
	II	93	1.86 (0.70, 4.93)	0.213
	III	41	5.97 (2.26, 15.80)	<0.001
	IV	10	12.59 (3.95, 40.14)	<0.001

B

Variable		N	Hazard ratio	p
**Subtype**	Luminal A	179	Reference	
	Luminal B	40	1.46 (0.82, 2.58)	0.2
	Her 2	31	1.49 (0.86, 2.58)	0.2
	Basal	84	4.45 (2.86, 6.94)	<0.001
**Stage**	I	71	Reference	
	II	161	1.36 (0.76, 2.44)	0.3
	III	80	3.47 (1.89, 6.36)	<0.001
	IV	22	7.61 (3.63, 15.94)	<0.001
**Age**		334	1.04 (1.02, 1.05)	<0.001

C

Variable		N	Hazard ratio	p
**CytoOnly.Pct**		174	0.97 (0.95, 0.99)	<0.001
**TotalCyto.Pct**		174	1.03 (1.01, 1.05)	0.003
**NucStainingInd**	Nuc−	145	Reference	
	Nuc+	29	0.21 (0.05, 0.85)	0.028
**Subtype**	Luminal A	98	Reference	
	Luminal B	22	0.49 (0.19, 1.28)	0.146
	Her 2	13	0.84 (0.33, 2.14)	0.713
	Basal	41	1.91 (1.04, 3.52)	0.038
**NPI**	Index 1	5	Reference	
	Index 2	26	0.68 (0.08, 5.79)	0.721
	Index 3	87	0.70 (0.08, 6.11)	0.748
	Index 4	56	1.52 (0.16, 14.44)	0.717
**Tumour.Size**		174	0.99 (0.97, 1.01)	0.394
**Stage**	I	41	Reference	
	II	92	1.37 (0.54, 3.44)	0.507
	III	41	2.74 (0.81, 9.26)	0.105
**Age**		174	0.99 (0.96, 1.03)	0.721
**Menopause**	Pre,Peri	66	Reference	
	Post	108	1.56 (0.63, 3.83)	0.333

A

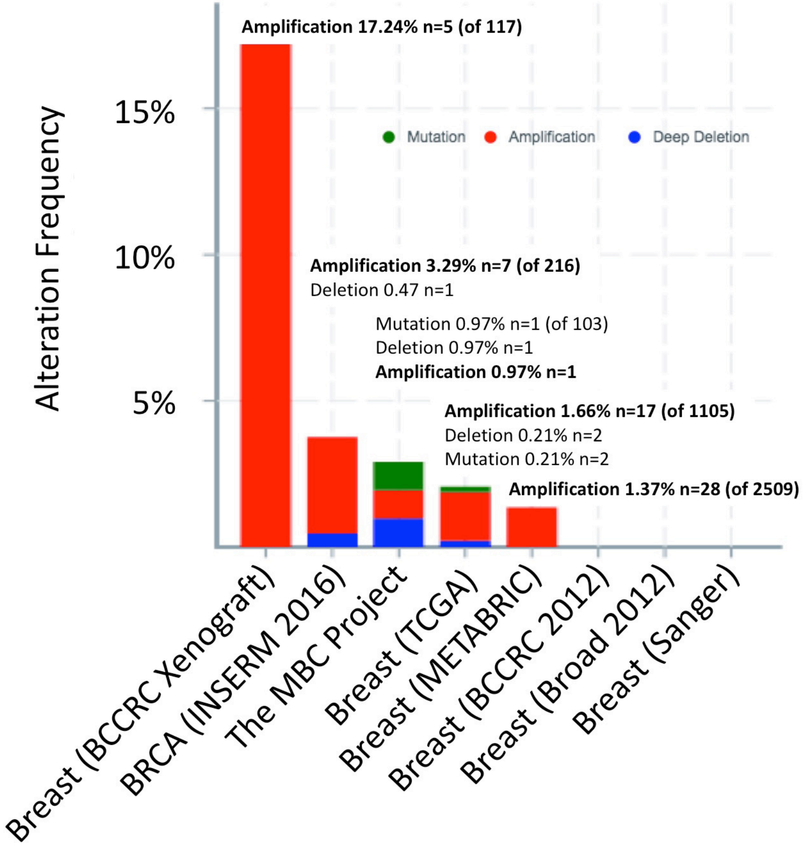

B

KAT5

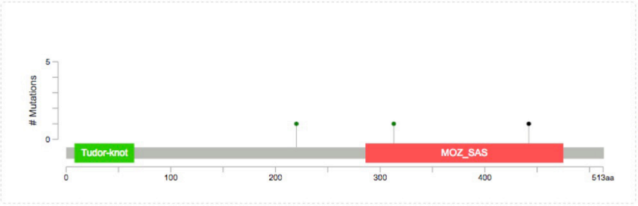

KAT5
UniProt: KAT5_HUMAN
Transcript: ENST00000377046
Somatic Mutation Frequency: 0.1%

2 Missense 1 Truncating
0 Inframe 0 Other

View 3D Structure

3 Mutations (page 1 of 1)

Columns

Study	Sample ID	Protein Change	Annotation ▼	Mutation Type	Copy #	COSMIC	Allele Freq (T)	# Mut in Sample
The Metastatic B...	MBC-MBCProject_5...	*S313F*		Missense	Gain			694
Breast Invasive ...	TCGA-A2-A0D1-01	*R220Q*		Missense	Diploid		0.21	58
Breast Invasive ...	TCGA-BH-A18P-01	*X442_splice*		Splice	ShallowDel		0.38	271

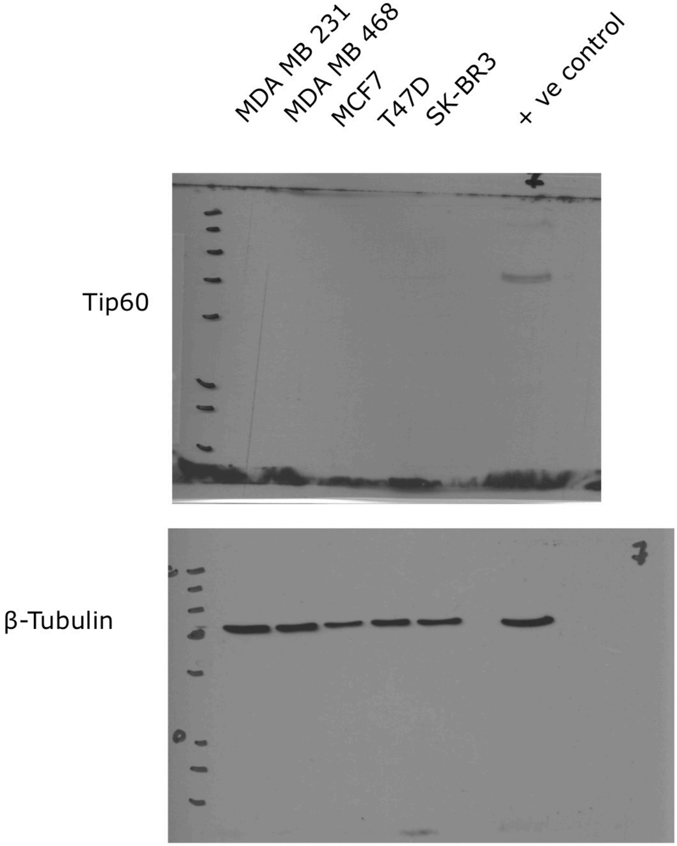
MDA MB 231
MDA MB 468
MCF7
T47D
SK-BR3
+ ve control
Tip60
β-Tubulin
